# A Crucial Role of Flagellin in the Induction of Airway Mucus Production by *Pseudomonas aeruginosa*

**DOI:** 10.1371/journal.pone.0039888

**Published:** 2012-07-02

**Authors:** Fatima Ben Mohamed, Ignacio Garcia-Verdugo, Mathieu Medina, Viviane Balloy, Michel Chignard, Reuben Ramphal, Lhousseine Touqui

**Affiliations:** 1 Unité de Défense Innée et Inflammation, Institut Pasteur, Paris, France; 2 INSERM U.874, Paris, France; 3 Department of Medicine, University of Florida, Gainesville, Florida, United States of America; French National Centre for Scientific Research, France

## Abstract

*Pseudomonas aeruginosa* is an opportunistic pathogen involved in nosocomial infections. Flagellin is a *P. aeruginosa* virulence factor involved in host response to this pathogen. We examined the role of flagellin in *P. aeruginosa*-induced mucus secretion. Using a mouse model of pulmonary infection we showed that PAK, a wild type strain of *P. aeruginosa*, induced airway mucus secretion and mucin muc5ac expression at higher levels than its flagellin-deficient mutant (ΔFliC). PAK induced expression of MUC5AC and MUC2 in both human airway epithelial NCI-H292 cell line and in primary epithelial cells. In contrast, Δ*FliC* infection had lower to no effect on MUC5AC and MUC2 expressions. A purified *P. aeruginosa* flagellin induced MUC5AC expression in parallel to IL-8 secretion in NCI-H292 cells. Accordingly, Δ*FliC* mutant stimulated IL-8 secretion at significantly lower levels compared to PAK. Incubation of NCI-H292 cells with exogenous IL-8 induced MUC5AC expression and pre-incubation of these cells with an anti-IL-8 antibody abrogated flagellin-mediated MUC5AC expression. Silencing of TLR5 and Naip, siRNA inhibited both flagellin-induced MUC5AC expression and IL-8 secretion. Finally, inhibition of ERK abolished the expression of both PAK- and flagellin-induced MUC5AC. We conclude that: (*i*) flagellin is crucial in *P. aeruginosa*-induced mucus hyper-secretion through TLR5 and Naip pathways; (*ii*) this process is mediated by ERK and amplified by IL-8. Our findings help understand the mechanisms involved in mucus secretion during pulmonary infectious disease induced by *P. aeruginosa,* such as in cystic fibrosis.

## Introduction

The Gram-negative bacterium *Pseudomonas aeruginosa* is an opportunistic human respiratory pathogen involved in a number of nosocomial infections [1], [2]. To protect its mucosal surfaces from infections by pathogens, the host uses sophisticated recognition systems including Toll-like receptors (TLRs) expressed by mucosal epithelial cells and macrophages, which sense conserved pathogen-associated molecular patterns (PAMPs) [3], [4], [5]. *P. aeruginosa* expresses numerous PAMPs [6] among which LPS and flagellin play a key role in host response to this bacterium, through interactions with TLR4 and TLR5, respectively [7]. Flagellin is a protein that assembles as a hollow cylinder with a cap to form the major portion of the bacterial flagellum [8]. Pre-clinical evidences showed that defects in TLRs or in downstream signalling pathways render the host susceptible to infection by pathogenic bacteria including *P. aeruginosa* [9]. The nucleotide-binding and oligomerization domain (NOD)-like receptor (NLR) family have been also identified as intracellular pattern recognition molecules for various microbial pathogens, including *P. aeruginosa* [10]. Human NLR family is composed of numerous pattern recognition molecules including NOD1, NOD2, Ipaf and Naip reviewed in [10]. To date, it is known that NOD1 and NOD2 interact with bacterial cell wall components, whereas Ipaf and Naip interact with bacterial flagellin [11]. Subsequent studies using *Salmonella* and *Legionella* suggested redundancy between Ipaf and Naip in the recognition of flagellin, but whether this finding can be extrapolated to other bacteria remains to be determined [11]. It has been shown that activation of NLRs leads to NF-kB or Caspase-1 activation, resulting in subsequent secretion of pro-inflammatory cytokines [11]. In contrast to Ipaf expression that is restricted to macrophages, Naip is expressed in both macrophages and lung epithelial cells [12].

Mucins are high-molecular weight and heavily glycosylated proteins, produced by the mucosal cells to protect the mucosal surface by trapping the inhaled infectious pathogens [13]. To date, 20 types of mucins have been identified [13], [14], [15], among them MUC5AC and MUC5B are important components of airway mucus in normal subjects [16]. MUC5AC and MUC5B are involved in the pathogenesis of respiratory infectious diseases, such as cystic fibrosis (CF) and chronic obstructive pulmonary disease, and contribute considerably to amplification of inflammation and tissue injury [13], [14], [15], [17], [18], [19]. MUC2, that is expressed normally by the intestinal epithelium, is highly elevated at the mRNA level in CF airways and following exposition with *P. aeruginosa* supernatant *in vitro* in NCI-H292 cells [20], [21]. In addition, increases in both MUC5AC and MUC2 mRNA levels have been reported in NCI-H292 cells after stimulation with *P. aeruginosa* culture supernatant through MAP kinase pathway [22], [23]. Our previous study showed that *P. aeruginosa-*derived LPS induced mucin expression [24], but the contribution of other PAMPs and their receptors in this expression has not been fully investigated. In addition, the mechanisms by which *P. aeruginosa* infection leads to mucus secretion in airway epithelial cells remain to be determined. The present work was undertaken to examine the role of flagellin in *P. aeruginosa*-induced mucus secretion and to determine the underlying mechanisms using human airway epithelial cells and a mouse model of acute lung infection.

## Results

### Flagellin is Involved in Airway Mucus Secretion in *P*. *aeruginosa* Infected Mice

**Figure 1:**
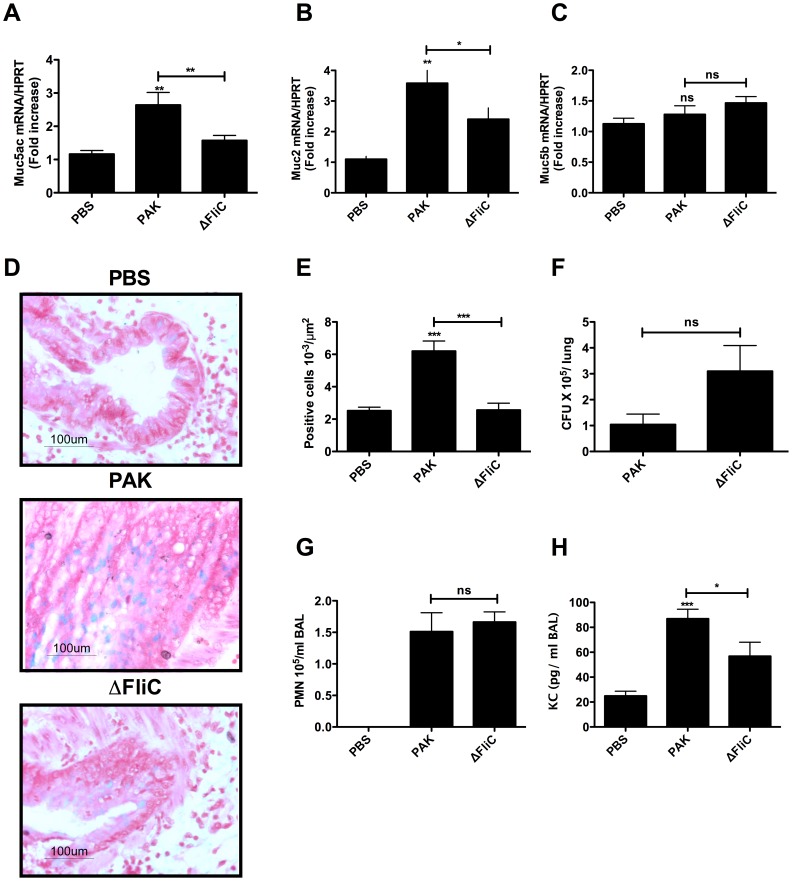
Effect of flagellin on mucus secretion and lung inflammatory response induced by *P. aeruginosa* infection. C57BL/6 female mice were infected for 24 hours with wild type *P. aeruginosa* strain (PAK) or its flagellin-deficient mutant (ΔFliC), (5 × 10^6^ CFU), as indicated in *Material* and *Methods.* (A) Shows muc5ac expression in lungs of PAK-infected compared to ΔFliC-infected mice and (B) Shows muc2 expression in lungs of PAK- and ΔFliC-infected mice. (C) Shows muc5b expression in lungs of PAK- and ΔFliC-infected mice. (D) Shows histological analysis with Alcian blue staining of the lung. The photographs were taken using a 40× magnification. The arrows point to mucus producing cells as stained by the blue (Alcian blue-positive cells). (E) Shows the number of Alcian blue-positive cells that were calculated from 3 independent experiments (5 mice in each group). (F) Shows bacteria amounts (CFU) in the lung detected 24 h following infection with 5 × 10^6^ CFU of either PAK- or ΔFliC. (G) Compares PMN influx in the lung of PAK- and Δ*FliC*-infected mice. (H) Shows the amount of KC pro-inflammatory cytokine produced in the lung of mice (n = 5), 24 h post-infection with 5 × 10^6^ CFU. nd  =  no detected, ns =  not significant. ***P<0,001, **P<0.01 when comparing to PBS; &&& *P*<0.001, && *P*<0.01 when comparing ΔFliC to PAK infected lungs.

Intranasal infection of mice with the wild type strain of *P. aeruginosa* (PAK) for 24 hours induced a 3-fold increase in the amount of pulmonary *muc5ac* mRNA in WT mice as compared to PBS-treated mice (Fig. 1A). In contrast, under similar conditions, infection of mice with the flagellin-deficient mutant (ΔFliC) did not lead to a significant increase in *muc5ac* expression. A significant difference in the level of *muc2* mRNA was also detected between PAK vs. ΔFliC lungs (Fig. 1B). However, no significant difference in the level of muc5b mRNA was detected in the lung of mice infected with PAK vs. ΔFliC (Fig. 1C). However, the number of mucus-positive cells, as assessed by Alcian Blue staining, was markedly decreased in the lung of mice infected with ΔFliC, as compared to mice infected with PAK (Fig. 1D and E). Interestingly, the decrease of mucins in the lung of mice infected with ΔFliC was not associated with a decrease in the replication of this mutant in the lung, because similar amount of bacteria was detected in PAK and ΔFliC lungs, 24 h post-infection (Fig. 1F). Similar amount of polymorphonuclear leucocyte (PMN) recruitment was also detected in PAK and ΔFliC lungs, 24 h post-infection (Fig. 1G). Interestingly, the decrease of mucins in the lung of mice infected with ΔFliC was associated with a decrease in the amount of KC produced in the lung following infection with the ΔFliC mutant (Fig. 1H). Altogether, these findings suggest that flagellin plays a critical role in stimulation of airway mucus secretion in *P. aeruginosa-*infected mice.

### *P. aeruginosa* Induces MUC5AC and MUC2 Expressions in Human Airway Epithelial Cells through a Flagellin-dependent Process

**Figure 2:**
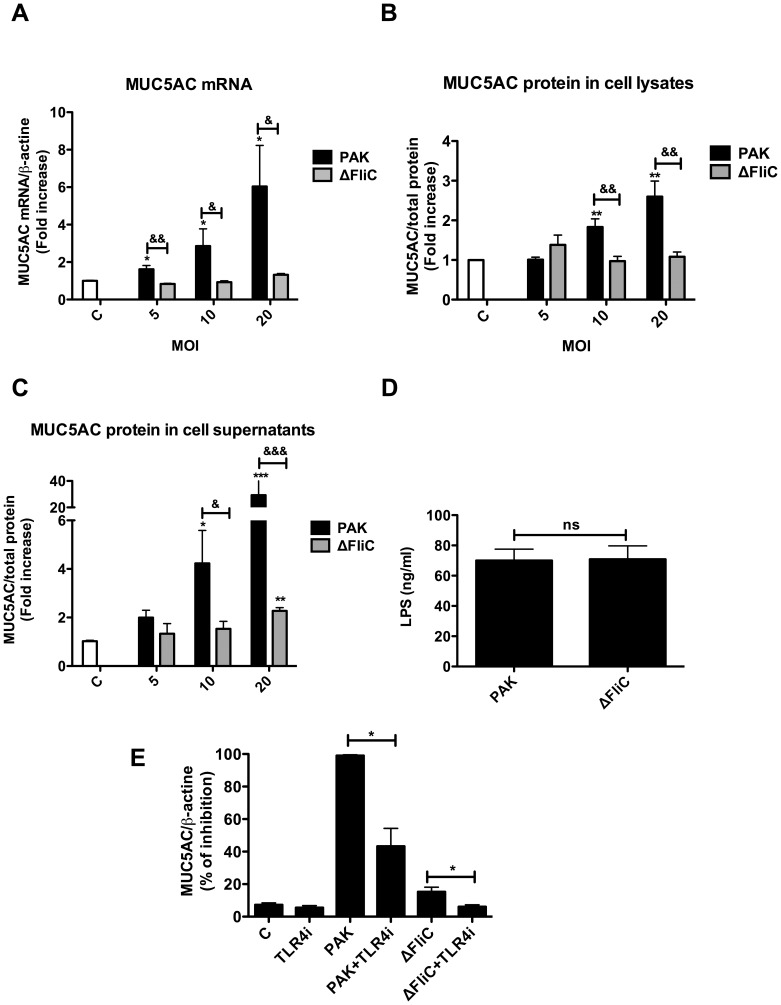
Lack of flagellin compromises *P. aeruginosa*-induced MUC5AC expression in NCI-H292 cells. (A) Cells were exposed to various MOI (5, 10 and 20 MOI) of the living PAK or ΔFliC strain for 24 h, then total mRNA was prepared and subjected to RT-qPCR using 18S as a standard. The levels of MUC5AC protein present in cell lysates (B) and in supernatants (C) were measured using an ELISA assay, as described in *Materials and Methods*. (D) Shows the levels of LPS in NCI-H292 supernatants after 1 h of infection with PAK or ΔFliC. (E) Shows the cells pre-treated with TLR4 inhibitor (TLR4i, 5 µM), as indicated in *Material* and *Methods.* before incubation with bacteria. The results are expressed as the percentage of inhibition of MUC5AC mRNA expression in TLR4i treated vs untreated cells. Values represent means ± SM of 3 independent experiments. *P<0.05, **P<0.01 and ***P<0.001 when comparing PBS to infected cells. C  =  control. & *P*<0.05, && *P*<0.01, &&& P<0.001 when comparing ΔFliC to PAK infected cells.

To investigate whether our finding can be extended to human airway mucus secretion, we studied the effect of *P. aeruginosa* infection on mucin expression by human airway epithelial cells, NCI-H292. Infection of these cells with PAK led to an increased MUC5AC expression both at mRNA (Fig. 2A) and protein levels (Fig. 2B). This was accompanied by MUC5AC secretion into culture medium (Fig. 2C). This induction was significant at PAK MOI as low as 5 (Fig. 2A). Interestingly, both MUC5AC expression and secretion were markedly reduced when cells were infected with the ΔFliC mutant. In contrast, similar levels of LPS were detected in the supernatants of NCI-H292 cells infected with PAK or ΔFliC strains (Fig. 2D). Incubation of NCI-H292 cells with the TLR4 inhibitor CLI-095 significantly reduced both PAK- and ΔFliC-induced MUC5AC expression (Fig. 2E).

**Figure 3:**
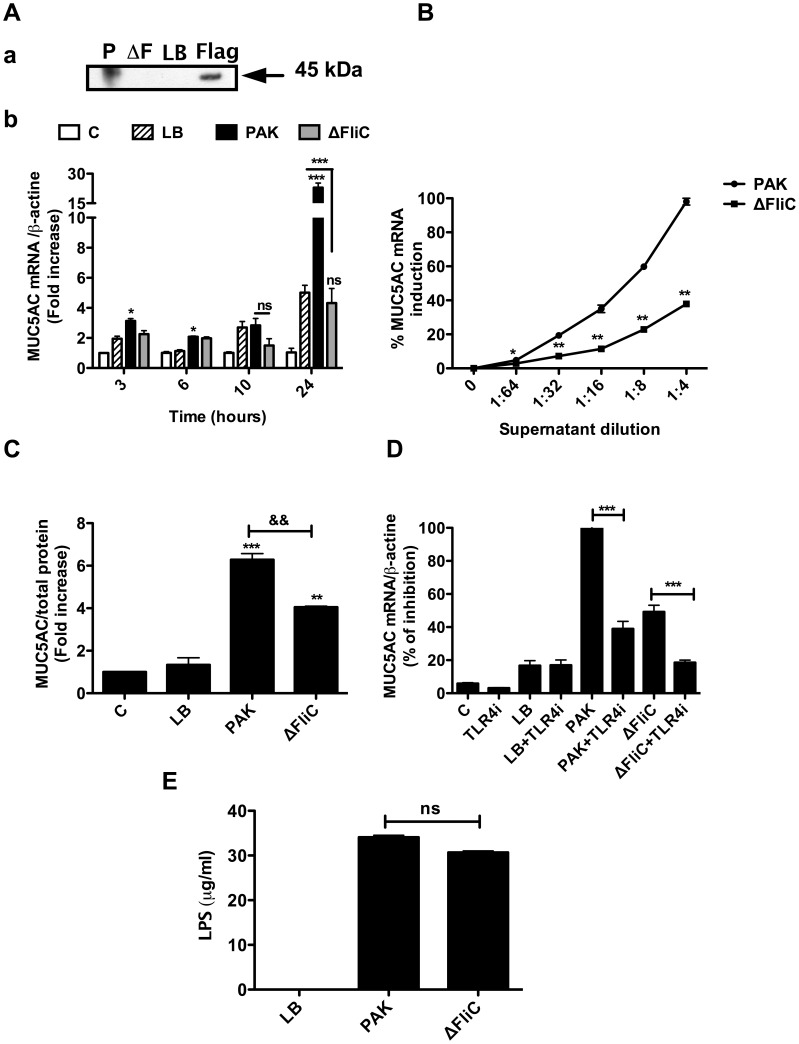
Lack of flagellin reduces *P. aeruginosa* supernatant-induced MUC5AC expression by NCI-H292 cells. NCI-H292 cells were exposed for 3, 6, 10 or 24 hrs to supernatants from either PAK or ΔFliC at 1∶8 dilution (A, b) or at the indicated dilution (B). The Insert (A, a) indicates immunoblotting analysis which shows the presence of flagellin in PAK (P) and its absence in ΔFliC (ΔF) supernatants. A 20 ng of purified flagellin from PAK (Flag) was used as a positive control and the bacteria culture medium (LB) was used as a negative control. Total mRNA was extracted and subjected to RT-qPCR (A, B). The level of MUC5AC protein produced in NCI-H292 supernatant was measured by ELISA, as indicated in *Material and Methods* (C). In (D) NCI-H292 cells were pre-treated with TLR4i (5 µM) one hour before incubation with bacterial supernatants from either ΔFliC or PAK and treated again during stimulation. The results show the percentage of inhibition of MUC5AC mRNA expression in TLR4i treated NCI-H292 cell compared to untreated NCI-H292 cells. E) Shows the LPS levels in ΔFliC vs. PAK bacterial supernatant after 24 h of culture. C  =  control (untreated cells), LB  =  Luria Bertoni medium 1∶8 dilution. Values represent means ± SM of 3 independent experiments. * *P*<0.05, ** *P*<0.01, *** *P*<0.001 *vs* Control (C); &&& *P*<0.001, && *P*<0.01 *vs.* corresponding PAK-treated controls or LB.

We next compared the effect of PAK and ΔFliC supernatants on mucin expression in NCI-H292 cells. For this experiment, we first ascertained that no flagellin was present in ΔFliC supernatant (Fig. 3Aa) and that this mutant released similar levels of LPS compared to PAK (Fig. 3E). Second, to determine the optimal time of mucin expression, we performed a kinetic study of mucin5AC expression in NCI-H292 cells stimulated with either PAK or ΔFliC supernatant, using 3, 6, 10 and 24 time points. Incubation of NCI-H292 cells with PAK supernatants led to an increased MUC5AC expression at both mRNA and protein levels (Fig. 3Ab, B and C). Our results indicate that the pic of expression of MUC5AC occurred at 24-hrs in NCI-H292 cells stimulated with PAK supernatant (Fig. 3Ab). Accordingly, all subsequent experiments were carried out using the time point of 24-hrs. In addition, the MUC5AC expression was significantly reduced in cells stimulated with ΔFliC supernatants (Fig. 3Ab, B and C). ΔFliC supernatants induced lower MUC5AC expression compared to PAK supernatants, regardless of the dilutions used (1∶64 to 1∶4) (Fig. 3B). A complete inhibition of ΔFliC supernatant-induced MUC5AC expression was achieved by incubating NCI-H292 cells with the TLR4 inhibitor (Fig. 3D).

**Figure 4:**
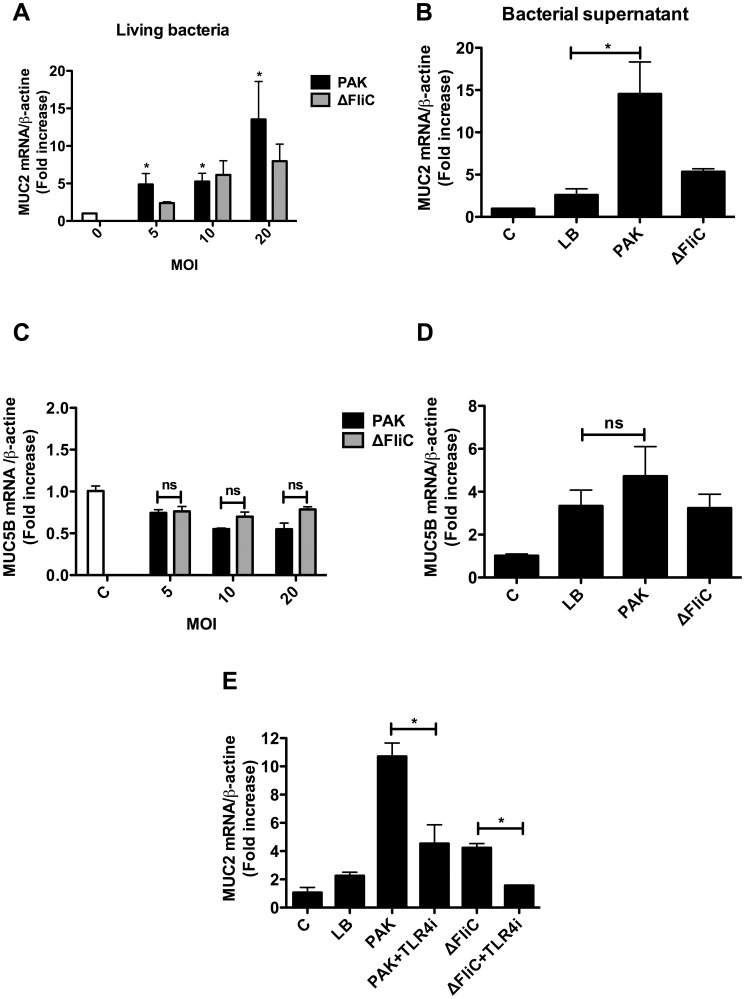
Deletion of flagellin abrogates *P. aeruginosa*-induced MUC2 expression in NCI-H292 cells. Cells were exposed to various MOI of living bacteria (A, C) or bacterial supernatants (B and D) as indicated in *Material* and *Methods*. Total mRNA was prepared and subjected to RT-qPCR to measure MUC2 (A, B) and MUC5B (C, D) levels. (E) NCI-H292 cells were pre-treated with TLR4i (5 µM) one hour and then incubated with bacteria supernatants. The result shows the percentage of inhibition of MUC2 mRNA expression following TLR4i treatment as compared to untreated control cells. Values represent means ± SM of 3 independent experiments. ns  =  no significant. C  =  control cells. * *P*<0.05 infected *vs.* no infected cells:

We also investigated whether flagellin is involved in the induction of two other mucins, MUC2 and MUC5B. The results showed that in comparison with living bacteria and supernatants from PAK, ΔFliC mutant led to a significant reduction in MUC2 mRNA expression (Fig. 4A, B). A complete inhibition of ΔFliC-induced MUC2 expression was achieved by incubating the cells with the TLR-4 inhibitor (Fig. 4E). Neither PAK nor ΔFliC mutant induced a significant level of MUC5B mRNA expression by NCI-H292 cells (Fig. 4C, D). The level of LDH released into the culture medium was less than 10% in both stimulated and unstimulated control NCI-H292 cells, and this level was similar regardless whether NCI-H292 cells were infected with PAK or ΔFliC (Fig. S1-A and Fig. S2-A). This result indicates that PAK and ΔFliC infections had no cytotoxic effect on of NCI-H292 cells. Furthermore, no change in the levels of total protein was observed following stimulation with PAK vs. ΔFliC (Fig. S1-B and Fig. S2-B) suggesting that no cell detachment occurred following PAK and ΔFliC infections. In addition, as indicated in Fig. 2E, there was no difference in LPS production detected in supernatant of cells stimulated with PAK vs. ΔFliC. Together, these findings suggest that flagellin: (*i*) is involved in both MUC5AC and MUC2 expressions in NCI-H292 cells; and (*ii*) is released by bacteria at sufficient amounts to induce these expressions.

### *P. aeruginosa* Induces Mucin Expression in Human Primary Bronchial Epithelial Cells

**Figure 5:**
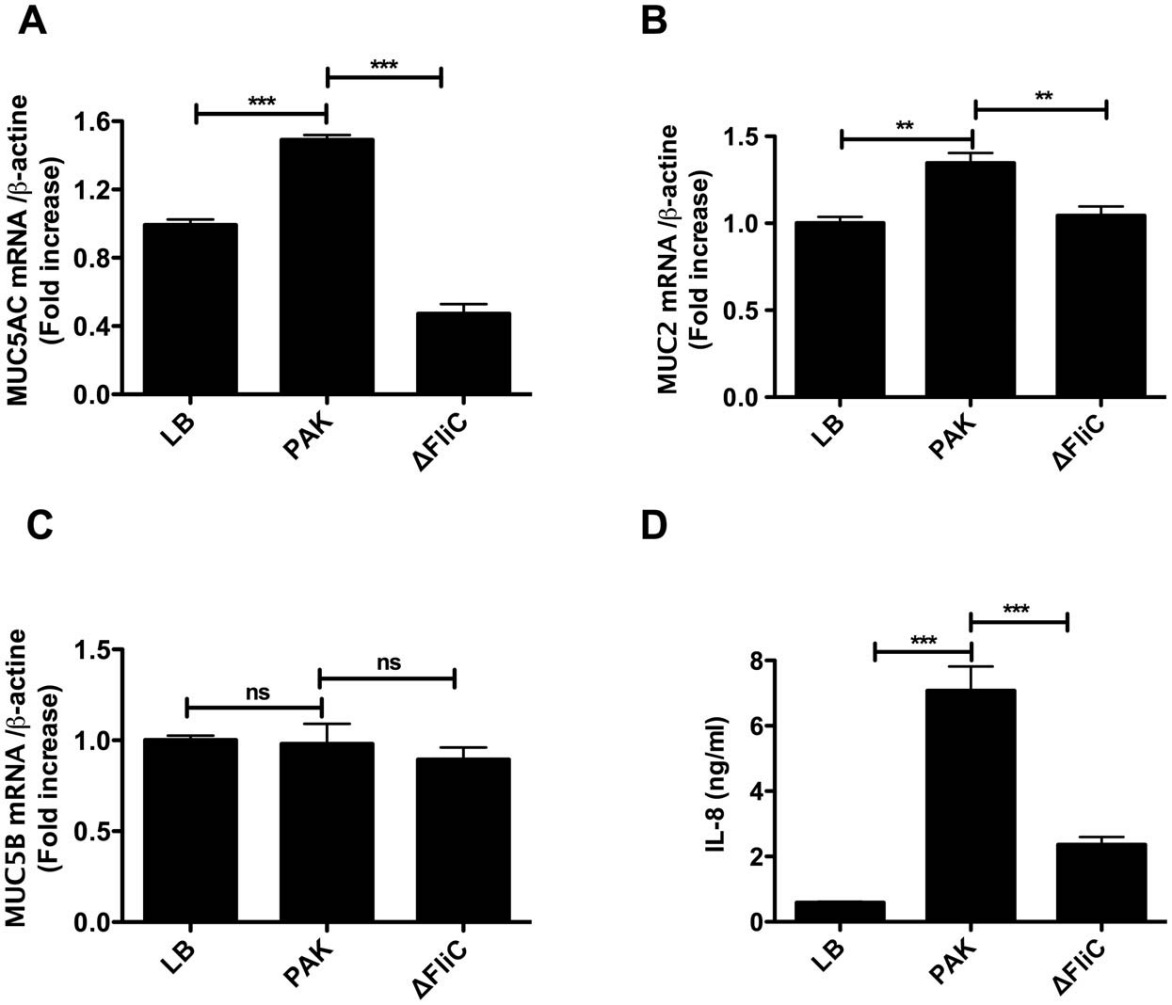
Lack of flagellin reduces *P. aeruginosa* supernatant-induced mucin expression in human primary bronchial epithelial cells. Human primary bronchial epithelial cells were exposed for 24 hours to supernatants from either PAK or ΔFliC at 1∶8 dilution. Total mRNA was prepared and subjected to RT-qPCR to measure MUC5AC (A), MUC2 (B) and MUC5B (C). (D) Shows the amount of IL-8 secreted into culture supernatants of human primary bronchial epithelial following stimulation with either PAK or ΔFliC supernatant, as detected by ELISA. The Values represent means ± SM of 3 independent experiments from three different donors done in replicates. ns  =  no significant, *P<0.05, **P<0,01, ***P<0,001 stimulated *vs.* unstimulated control cells.

We next investigated whether our findings can be extended to human primary bronchial epithelial cells. These cells were differentiated in air-liquid interface and stimulated with supernatants from either PAK or ΔFliC. PAK supernatants induced increased MUC5AC and MUC2 expressions in human primary bronchial epithelial cells (Fig. 5A and B). Although these expressions occurred at lower levels in human primary bronchial epithelial cells as compared to the levels observed in NCI-H292 cells there was significantly reduction in cells stimulated with ΔFliC vs. PAK supernatants. In agreement with the results obtained with NCI-H292 cells, neither PAK nor ΔFliC supernatant were capable of inducing MUC5B mRNA expression (Fig. 5C). In addition, the level of expression of the pro-inflammatory cytokine IL-8 was significantly reduced in human primary bronchial epithelial cells stimulated with ΔFliC supernatant compared to cells stimulated with PAK supernatant (Fig. 5D).

### Purified *P. aeruginosa* Flagellin Induces MUC5AC Expression through TLR5 and NAIP Receptors

**Figure 6:**
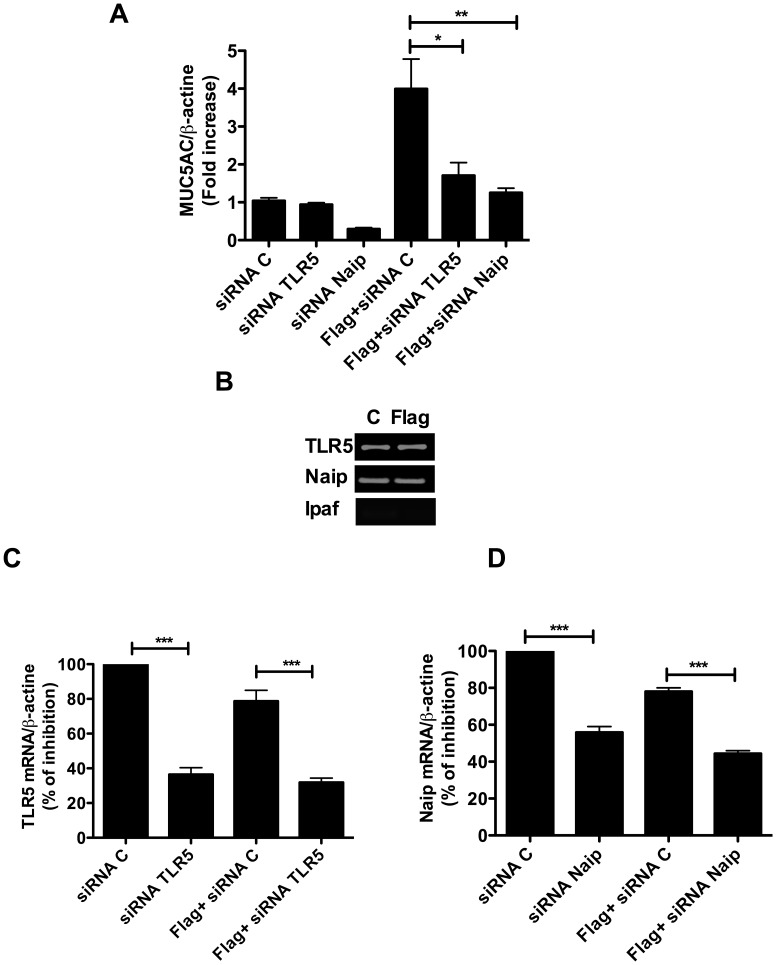
Impact of TLR5 and NAIP silencing on MUC5AC expression. NCI-H292 cells were transfected with TLR5 or Naip siRNA and then treated with or without purified flagellin (Flag, 1 µg/ml) for 24 h. Scrambled siRNA was used as a negative control for transfection. (A) Shows the effect of siRNA transfection on flagellin-induced MUC5AC expression. (B) Shows TLR5, Naip and Ipaf expression in NCI-H292 analyzed by RT-PCR. (C) and (D) show the efficiency of siRNA transfection on TLR5 and Naip expressions compared to siRNA controls (C). Data are expressed as the means ± SM. P<0,05. * *P*<0.05, ** *P*<0.01, *** *P*<0.001 *vs* Controls, 3 independent experiments.

The findings depicted above led us to examine the effect of purified *P. aeruginosa* flagellin on mucins expression in epithelial cells. Our results showed that purified flagellin was sufficient to induce MUC5AC mRNA expression in epithelial cells (Fig. 6A). This induction cannot be attributed to contamination by LPS, because the latter was found at a low concentration in our flagellin preparation(i.e. below 1 pg/ml). We showed that at least 100 ng/ml of LPS was necessary to induce MUC5AC expression in NCI-H292 cells (data not shown). We next examined the role of flagellin receptors TLR5, Naip and Ipaf in flagellin-induced MUC5AC expression. We showed that both TLR5 and Naip, but not Ipaf, were expressed in NCI-H292 cells (Fig. 6B). Silencing of TLR5 and Naip reduced flagellin-induced MUC5AC mRNA levels by 57% and 70%, respectively, compared to siRNA controls (Fig. 6A). We verified that TLR5 and Naip siRNA treatments reduced the levels of TLR5 and Naip by 70% and 50%, respectively (Fig. 6C and D). These findings suggest that both TLR5 and Naip play a role in flagellin-induced MUC5AC expression in NCI-H292 cells.

### Flagellin Induces MUC5AC Expression Partially through IL-8 Secretion by NCI-H292 Cells

**Figure 7:**
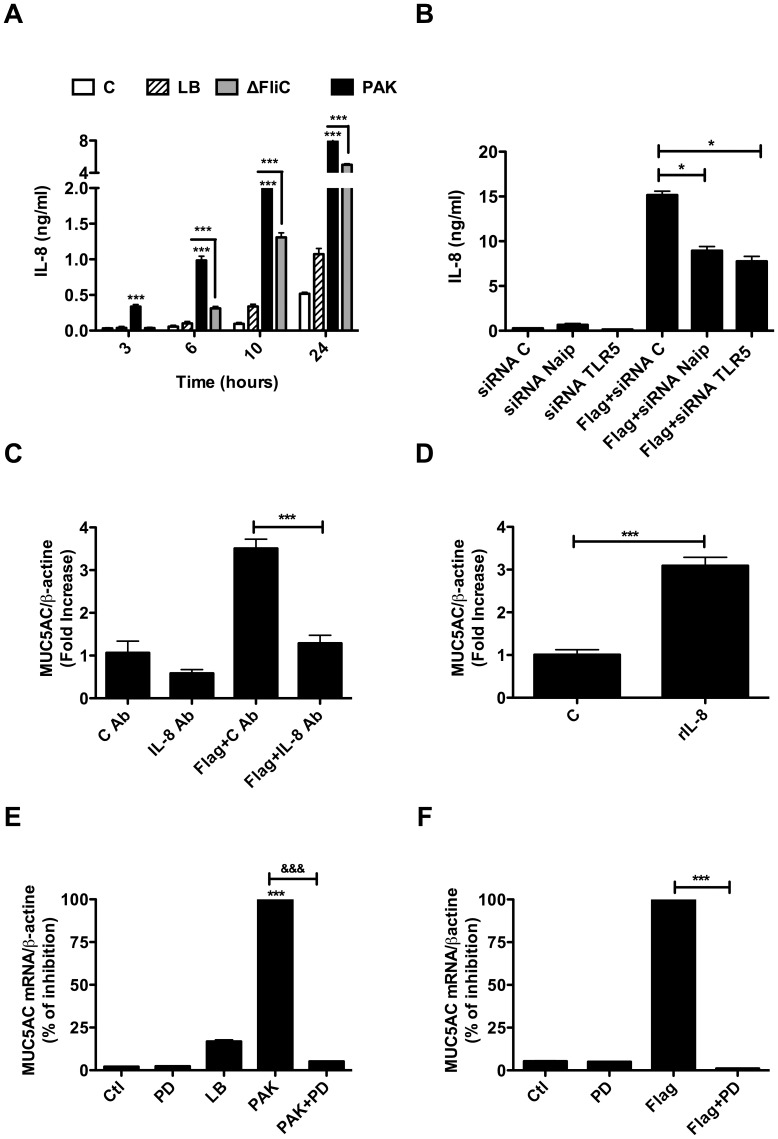
*P. aeruginosa* flagellin induces MUC5AC expression through IL-8 signalling. (A) NCI-H292 cells were exposed to PAK- or ΔFliC bacteria supernatant for 3, 6, 10 or 24 hrs and the IL-8 secreted into culture supernatants was measured using an ELISA, as described in *Materials and Methods*. (B) Shows IL-8 secretion by NCI-H292 cells after transfection by control siRNA (C), Naip siRNA or TLR5 siRNA with or without stimulation with flagellin (at 1 µg/ml). (C) Shows MUC5AC expression by NCI-H292 cells after incubation with flagellin with anti-IL-8 neutralizing or isotype control antibody. (D) Shows Induction of MUC5AC mRNA expression in NCI-H292 cells exposed to a rIL-8 (at 10 ng/ml). (E, F) shows the effect of ERK1/2 inhibitor PD98059 (20 µM) on MUC5AC expression by NCI-H292 cells stimulated with PAK supernatant or with purified flagellin. Values represent means ± SM of three independent experiments. * P<0.05, ** *P*<0.01, *** *P*<0.001 *vs.* LB.

Infection of NCI-H292 cells by PAK led to a time-dependent increase of IL-8 secretion compared to ΔFliC (Fig. 7A). Similar result was obtained when primary cells were stimulated with bacterial supernatant (Fig. 5D). Our results also showed that purified flagellin induced IL-8 secretion and that silencing of TLR5 and Naip expressions significantly inhibited this secretion (Fig. 7B). We next examined the possible role of IL-8 in MUC5AC expression in flagellin-stimulated cells. Pre-incubation of NCI-H292 cells with an IL-8 neutralizing antibody significantly decreased flagellin-induced MUC5AC expression (Fig. 7C) compared with control antibody. In addition, stimulation of cells with a recombinant IL-8 induced a three-fold increase in MUC5AC mRNA levels (Fig. 7D). Finally, the expression of MUC5AC in NCI-H292 cells stimulated by either PAK supernatant or flagellin was abolished following MEK1/2 signalling pathway inhibition (Fig. 7E, F). However, treatment of NCI-H292 cells with a P-38 signalling pathway inhibitor had no effect on MUC5AC expression (data not shown). These findings indicate that flagellin induced MUC5AC expression in part through IL-8 secretion and that this process is mediated by MEK1/2 signalling pathway.

## Discussion

The present study demonstrated that flagellin plays a role in *P. aeruginosa-*induced airway mucus secretion. Indeed, ΔFliC, the flagellin-deficient mutant of *P*. *aeruginosa* induced mucin expression and mucus secretion at much lower levels than the corresponding wild type PAK strain, both in a mouse model of pulmonary infection and in human bronchial epithelial cells, both primary and cell line. Interestingly, our findings showed that bacterial supernatants were able to induce mucin expression and that this ability was markedly reduced in ΔFliC supernatants. This suggested that flagellin is released in the incubation medium of bacteria at sufficient amounts to induce mucin expression. This was supported by the fact that *P. aeruginosa* flagellin stimulated mucin expression by human airway epithelial cells. Interestingly, the present studies also showed that flagellin is involved not only in the induction of MUC5AC but also in MUC2 expression. Taken together, these findings suggest that flagellin plays a role in the induction of airway mucus secretion during *P. aeruginosa* lung infection and that this process might have a pathophysiological significance for lung diseases.

One may argued that the reduced ability of the ΔFliC mutant to induce mucin expression might be due to the inability of this mutant to effectively infect the mouse lung. This is unlikely because our previous studies have clearly shown that flagellin deletion did not reduce lung infection by this bacterium, but rather increased it [25]. Indeed, in these studies ΔFliC exhibited slower clearance from the lung compared to PAK strain in a mouse model of acute lung infection [25]. This increase of infection with flagellin-deficient bacteria was observed not only with *P.* *pseudomonas* but also with *L. pneumophila* [12]. In addition, in our *in vitro* studies, reduced mucin expression by Δ*FliC* may not be due to altered ability of this mutant to adhere to epithelial cells because the latter were centrifuged after infection to achieve maximal adherence of bacteria. Moreover, no flagellin was detected in Δ*FliC* supernatant in which the levels of LPS were similar to those found in PAK supernatant, indicating that the decreased mucin expression induced by ΔFliC is clearly a consequence of lack of flagellin in this strain.

It has been shown that *P. aeruginosa* induces MUC5AC secretion by human airway epithelial cells through an LPS/TLR4 dependent mechanism [22], [26]. To determine whether, in our system, the LPS/TLR4 pathway is involved in mucin expression, we used a pharmacological inhibitor of TLR4, CLI-095. Unfortunately, LPS-deficient *P. aeruginosa* mutant was not available because knocking LPS is lethal in *P. aeruginosa.* This pharmacological inhibition reduced significantly mucin expression induced by both PAK and Δ*FliC*. This result also confirm our recent finding indicating that *P. aeruginosa* LPS exacerbates mucus secretion in a mouse model of CF [24]. Our results are also in agreement with a recent study by Sun *et al*., showing that *P. aeruginosa* induced-keratitis occurs through a TLR4/5 dependent mechanism [27]. The present report also shows that the pharmacological inhibition of TLR4 pathway during incubation with PAK supernatants led to a partial inhibition of mucin expression by epithelial cells. However, TLR4 inhibition completely abolished ΔFliC supernatant-induced-mucin expression. These findings suggest that LPS and flagellin may act synergistically or additively to induce mucin expression by epithelial cells. They also suggest that LPS and flagellin were released by the bacteria, at sufficient amounts, that stimulated mucin expression by epithelial cells. However, one should kept in mind that other PAMPs, such as pili and bacterial DNA may also play a role in the induction of mucin in epithelial cell following P. *aeruginosa* infection.

We next focused on the mechanisms by which flagellin induce mucins expression in cultured human epithelial cells. In addition to TLR5, flagellin is known to interact with Ipaf as well as with Naip intracellular receptors [12]. Previous finding showed that human Naip is expressed in both macrophages and lung epithelial cells, in contrast to Ipaf expression that was restricted to the macrophages [12]. Consistent with the above finding, our results demonstrate that TLR5 and Naip, but not Ipaf, are expressed in the human lung epithelial cells, NCI-H292. Given that Naip and Ipaf have been previously suggested to exert redundant role in the recognition of flagellin [28], Ipaf could compensate the absence of Ipaf in our cell system. Using siRNA silencing strategy to knockdown TLR5 and Naip, in lung epithelial cells, we showed that purified flagellin induced MUC5AC expression through both TLR5 and NAIP-dependent processes. A role of Naip-interaction with flagellin was observed in the activation of inflammasome-mediated innate immunity in mouse macrophages but not in epithelial cells [29], [30]. Interestingly, in lung epithelial cells, Naip knockdown led to an enhanced replication of wild-type *L. Pneumophila,* but not flagellin-deficient strain, suggesting the crucial role of this receptor in the flagellin interaction and bacteria replication [12]. In addition, in mice macrophages, Naip-5 specifically interacted with *L. Pneumophila* flagellin to induce caspase-1 activation and IL-1β cytokine secretion [30]. In our human lung epithelial cells model, neither the caspase-1 activation nor the IL-1β secretion was detected after stimulation with *P. aeruginosa* flagellin (Fig. S3). This suggests that the mucin expression induced flagellin may be triggered through an IL-1β-independent mechanism.

In a subsequent investigation we examined the possible relationship between flagellin-induced mucin expression and pro-inflammatory cytokine IL-8 secretion. Our results demonstrated that IL-8 secretion was induced by *P. aeruginosa* partially *via* a flagellin-dependent process. Indeed, stimulation of NCI-H292 cells with ΔFliC mutant induced a significantly lower secretion of IL-8 compared to the PAK strain. Similar results were obtained regardless of whether the cells were stimulated with the whole live bacteria or with their supernatants. This result confirmed our previous findings showing a lower level of keratinocyte-derived cytokine *(*KC), the mouse orthologue of IL-8, in both broncho-alveolar lavage and primary epithelial cells from mice infected with the ΔFliC mutant [7], [31]. Our data are also in agreement with the pervious reports showing that incubation of human airway epithelial cells with flagellin purified from *P. aeruginosa* induced IL-8 secretion [32]. Similarly to MUC5AC, flagellin-induced IL-8 secretion was also reduced by siRNA silencing of TLR5 and Naip. Interestingly, we showed that IL-8 mediates the effect of flagellin on MUC5AC expression. This was based on our finding that a neutralizing anti-IL-8 antibody reduced flagellin-induced MUC5AC expression in NCI-H292 cells. In addition, our results indicated that recombinant IL-8 induced a significant increase in MUC5AC expression, confirming a previous study showing that IL-8 stimulates MUC5AC and MUC5B expression in NCI-H292 at the posttranscriptional level [33]. It has been also shown that intratracheal instillation of murine recombinant chemoattractant chemokines MCP-1, MCP-5 and KC induced mucin expression in mouse lung [34]. However, these findings do not imply that these chemokines are the only ones involved in mucin expression. Other, yet to be determined, chemokines might also be involved in this process.

In conclusion, we reported that *P. aeruginosa* flagellin plays a critical role in the induction of airway mucus secretion both in cultured human bronchial epithelial cells and in a mouse model of lung infection by *P. aeruginosa*. This induction occurs via a process involving TLR5 and Naip receptors, mediated by MEK1/2 signalling pathway and amplified by IL-8. Our findings would help design future therapeutic strategies to reduce airway mucus secretion in chronic inflammatory lung disease such as CF.

## Materials and Methods

### Bacterial Strains and Growth Conditions

The *P. aeruginosa* wild type (WT) PAK strain, a widely studied strain of *P. aeruginosa* and its aflagellated Δ*FliC* mutant in which the *FliC* gene encoding flagellin was deleted [35] were prepared as indicated previously [7]. For the bacterial supernatant, the WT PAK and Δ*FliC* mutant were grown in LB medium for 24 h at 37°C. At equal quantity of bacteria, cell-free supernatants were obtained by centrifugation at 4000 g for 15 min at 4°C and then filtered through a 0.22 µm filter (Millipore, Molsheim, France). All supernatants were aliquoted and stored at −80°C until used.

### Mouse Infections

Females wild type (WT) C57BL/6 mice ∼8 wk of age were used the *in vivo* studies (Janvier Laboratories, France). Mice were fed normal mouse chow and water *ad libitum* and were bred and housed under standard conditions with air filtration. All animal studies were approved by the Pasteur Institute Safety Committee in accordance with French and European animal welfare regulations and guidelines. Mice were anaesthetized by i.m. adminstration of a mixture of ketamine (40 mg/Kg) and xylazine (8 mg/Kg) and infected intranasally with bacterial doses equivalent to approximately one-tenth of the LD_50_ of wild-type PAK strain (i.e. 5×10^6^ colony-forming unit (CFU) per mouse), as described previously [25].

### Flagellin Preparation and LPS Quantification

Type A *P. aeruginosa* flagellin was prepared and purified as described elsewhere [36]. To remove contaminating lipopolysaccharide (LPS), the purified flagellin was passed through a polymyxin B column, resulting in flagellin preparations with low levels of LPS (<1 pg/µg flagellin). We also quantified the levels of LPS in the supernatants of bacteria and NCI-H292 cells. Analysis of LPS levels was performed using the Limulus amebocyte lysate kit (Lonza, Basel, Switzerland). Flagellin protein purity was determined by SDS-PAGE and Coomassie blue staining. Purified proteins were quantified by the Bio-Rad protein assay (Bio-Rad, Marnes-la-Coquette, France).

### Alcian Blue/safranin Staining

Twenty-four hours post-infection, mice were euthanized following an i.p. injection of an overdose of pentobarbital sodium (300 mg/Kg). The chest was opened and lung perfused with sterile PBS through the pulmonary artery to remove circulating blood. In one series of experiments, the lung tissue was excised and immediately fixed in Carnoy’s fixation (60% ethanol, 30% chloroform and 10% glacial acetic acid). Tissue samples were then embedded in paraffin and five-µm-thick sections were then cut and mounted onto microscope slides for alcian blue/safranin staining. Alcian blue (AB) powder (0.1 g) was dissolved in 100 mL of acetic acid 3%. Concentrated HCl was added to lower the pH value of the solution to 5. Sections were stained for 30 min followed by washing with water. Sections were then stained using 1% safranin for 30 min, rinsed with ethanol, cleared in xylene and mounted with Pertex mounting medium (*Histolab* Products Ab., Gothenburg, Sweden). The number of Alcian blue-stained cells was measured. We performed analyses using MIRAX program with help of histology department of Institut Pasteur. Images were taken with a Nikon Eclipse E800 microscope (Nikon Corp.) and were acquired using a Nikon Eclipse DXM1200 digital camera (mounted on the Nikon Eclipse E800) and the Nikon ACT-1 software.

### *In Vitro* Infection of Human Airway Epithelial Cell Cultures with *P. aeruginosa*

Human epithelial NCI-H292 cells, obtained from American Type Culture Collection (ATCC, Manassas, USA) were cultured in RPMI-1640 medium supplemented with 200 mM L-glutamine, 10% (v/v) fetal calf serum, 100 UI/ml penicillin, 100 µg/ml streptomycin, 2.5 mg/l glucose and buffered with 25 mM HEPES at 37°C in a humidified, 5% CO_2_ water-jacketed incubator. For stimulation purposes, NCI-H292 cells were seeded into 24-well tissue-culture plates and grown until confluence. Cell stimulations and infections were performed in free serum culture medium to maintain a low basal level of mucins, as we [37] and others [38] have recently described. Cells were suspended in the free serum culture medium without antibiotics and stimulated with different multiplicities of infection (MOI) of bacteria, as specified in the figure legends. In order to increase the adherence between cells and bacteria and to ensure similar contact between cells, PAK and its non-motile Δ*FliC* mutant, infected cells were centrifuged (80 g, 4 min, 4°C). After one hour of incubation the bacteria were removed and cells were washed three times in 40 µg/ml of tobramycin medium (Sigma-Aldrich, Saint Quentin Fallavier, France). Cells were then incubated with tobramycin medium for 30 min at 37°C and washed with the same medium three times to ensure that all extracellular bacteria were removed or killed. Cells were then incubated overnight in RPMI medium with 0.1% fetal bovine serum and 1% antibiotic solution. In one series of experiments, cell cultures were stimulated following an 18 hrs exposure to several dilutions of bacteria supernatant as indicated in figure legends in 0.1% serum-medium and compared with LB, used as a control. In one series of experiments, cell cultures were incubated with the TLR4 inhibitor, CLI-095 (Invivogen, San Diego, USA) at a final concentration of 5 µM one hour before and during stimulation with living bacteria or their supernatants.

### *In Vitro* Stimulation of Human Airway Epithelial Cell with Flagellin

In one series of experiments, cell cultures were stimulated with 1 µg/µl of flagellin for 24 hours. Cells were pre-incubated with an anti-human CXCL8/IL-8 monoclonal antibody 5 µg/ml (R&D System, Lille, France) one hour before and during the stimulation with flagellin. In one series of experiments, the cells were stimulated with human recombinant IL-8, 10 ng/ml (R&D Systems, Lille, France),

### Examination of Flagellin Secretion by Immunoblotting

Flagellin release was examined by immunoblotting of the supernatants of bacteria grown at 37°C. At equal quantity of bacteria, the cell-free supernatants from WT PAK and ΔFliC mutant strains were diluted in RIPA buffer at 1/50. The purified *P. aeruginosa* flagellin was used at 20 ng as a positive control. The samples were then run on 15% native polyacrylamide gels and transferred to nitrocellulose membranes for immunoblotting using a non-commercial polyclonal rabbit antibody specific to *P. aeruginosa* flagellin, that was recently described by Arora et *al*. [39].

### Incubation of Differentiated Human Primary Bronchial Epithelial Cells with *P. aeruginosa* Supernatant

Differentiated human primary bronchial epithelial cells, MucilAir, were purchased from Epithelix **(**Epithelix Sarl, Geneve, Switzerland). These cells were isolated from the bronchi of healthy subject and cultured at air-liquid interface for 3 weeks in mucilAir culture medium (Epithelix) until differentiation. To reduce basal levels of mucin expression, cells were cultured for 48 h in BEBM basal medium (Lonza, CC-3171) supplemented with antibiotics. The cells were then stimulated in the same medium with bacteria supernatants or a equivalent dose of bacterial growth medium (LB) and 24 h after RNA extractions were performed.

### siRNA Transfection

ON-TARGET plus control siRNA, TLR5 and Naip siRNA were purchased from Dharmacon (Abgen, UK). NCI-H292 cells were incubated overnight in RPMI-1640 medium containing 10% FBS so that cells were 60% confluent at the time of transfection. Transfection was performed in OPTIMEM medium using Lipofectamin 2000 (Invitrogen, Cergy Pontoise, France) as transfection reagent and 100 nM final concentration of siRNA. After 8 hours, transfection media was removed and new complete media was added. Twenty-four hours after transfection, cells were stimulated with 1 µg/ml of flagellin for an additional 24 hours. Finally, cells were assayed for inhibition of targeted gene. In one series of experiments, to neutralize IL-8, the anti-human CXCL8/IL-8 antibody (R&D system, Lille, France) was incubated at 5 µg/ml with cells 1 hour before and during the stimulation with flagellin.

### Real-time Polymerase Chain Reaction

Total RNA was extracted from cultured NCI-H292 cells or from mice lung homogenate by RNeasy Mini Kit according to manufacturers’ instructions (Qiagen, Courtaboeuf, France). One µg of RNA was treated with recombinant RNAse-free DNAse I (Roche, Meylan, France) and then, the corresponding cDNA was synthesized using random hexamers (Roche, Meylan, France) and M-MLV reverse transcriptase (Promega, Charbonnières-les-Bains, France). Real-time polymerase chain reaction (RT-PCR) was performed using an ABI 7900 RT-PCR detection system (Applied Biosystems, Foster City, CA) in 10 µl reactions that contained 1 µl of diluted cDNA, 300 nM each of forward and reverse primer, and SYBR Green PCR Master Mix (Fisher scientific, Illkirch, France). Each sample was run in duplicate for each gene and the relative quantity (RQ) of mRNA was calculated based on the housekeeping gene. Ct values were determined using Microsoft Excel and the comparative Ct (ΔΔCt) method, as described by the manufacturer (Applied Biosystems). The amount of target (2^−ΔΔCT^) was normalized to house keeping gene, using control cells as calibrator (arbitrary units = 1), unless stated otherwise. The primers for MUC5AC, MUC2 and MUC5B have been previously described [40]. The primer for human β-actine has been described [37]. Other primers were designed using the Oligo Explorer 1.1. 2 software. The following is the sequence of these primers: human TLR5 (Fw:5′- TTCAACTTCCCAAATGAAGGA-3′; Rv:5′- TTGCATCCAGATGCTTTTCA-3′), human Naip (Fw:5′- CTTCACCCTGTGCCATTTCT-3′; Rw:5′- AGAGCCGTGGTGAACTTTGT-3′), human Ipaf (Fw:5′-ACCCAAGCTGTCAGTCAGACC-3′; Rw:5′- CCAGTCCCCTCACCATAGAAG -3′), mouse muc5ac (Fw:5′- ACGGATGCTGCTATGACTG-3′; Rv:5′- TGTGTGCTCTACCTTGTTGG-3′), mouse muc2 (Fw:5′-CGACACCAGGGCTTTCGCTTAAT-3′; Rv:5′-CACTTCCACCCTCCCGGCAAAC-3′), mouse muc5b (Fw:5′-CATAGCCACTGCTCTTCT-3′; Rv:5′-CTGGCCTCTGTAACTATGT-3′), mouse Hypoxanthine–Guanine Phosphoribosyltransferase *(*Hprt*)* (Fw:5′- CAGGCCAGATTTGTTGGAT-3′; Rv:5′- TTGCGCTCATCTTAGGCTTT-3′).

### ELISA Assay

MUC5AC ELISA was performed as previously described [41]. Briefly, 50 µl of cell lysate or 95 µl of culture supernatant were diluted with carbonate/bicarbonate buffer (0.05 M final concentration) and allowed to dry for at least 24 h in wells of a Maxisorb (Nunc) 96-well plates at 40°C. Wells were washed 3× with sterile PBS and blocked with PBS/2% BSA (fraction V) before adding 100 µl of anti-MUC5AC mAb diluted to 1/600 (clone 45M1) (Neomarkers Ab, Interchim, Montluçon, France) in PBS/1% BSA/0.1% Tween-20 for 1 h at room temperature. The plate was then washed with PBS and further incubated with peroxidase-conjugated goat anti-mouse IgG (1/10.000 in PBS/1% BSA/0.1% Tween-20) (Sigma-Aldrich, Saint Quentin Fallavier, France) for 1 h at room temperature. Peroxidase activity was detected using 3,3′,5,5′-tetramethylbenzidine solution and stopped with 2N H_2_SO_4_. Absorbance was read at 450_nm_ OD. Because secretion of MUC5AC protein by NCI-H292 cell changes with cell passages [41] and that there is no commercial standard available for human MUC5AC, its expression is represented as fold increase referred to the basal secretion (control untreated NCI-H292 cells) tested under same conditions. Total protein from cell lysates was quantified by MicroBCA protein assay (Thermo scientific Pierce, Illkirch, France) in order to eliminate effects of the stimuli on cell proliferation.

Interleukin-8 (IL-8) secretion were measured in supernatants using a human IL-8 Kit DuoSet sandwich ELISA (R&D Systems, Lille, France), following manufacturers’ instructions.

### Cytotoxicity Assay

Lactate dehydrogenase (LDH) activity was measured in the supernatants of all culture using a cytotoxicity detection kit (Roche), according to the manufacturer’s protocol. Results are represented as percentage of LDH released into the supernatant ((supernatant/supernatant + lysate) ×100 =  % LDH release). The treatment is considered not cytotoxic with less than 10% of LDH.

### Statistical Analysis

Data were represented as means ± S.E. and compared using the unpaired Student’s *t* test for the *in vitro* experiments and One-Way ANOVA test for the *in vivo* experiments using Newman-Keuls as secondary test to compare individual groups. *P* values less than 0.05 are considered significant.

## Supporting Information

Figure S1 **Infection of NCI-H292 cells with PAK and ΔFliC bacteria results in no cytotoxic effect.** (A) Shows LDH level released in supernatants of NCI-H292 epithelial cells (determined as % of total cell LDH) following infection with either PAK or ΔFliC living bacteria. (B) Compares the level total proteins detected in adherent NCI-H292 epithelial cells following stimulation with either PAK or ΔFliC living bacteria. (C) Compares the amount of bacteria (CFU) detected in NCI-H292 cell supernatants after the first hour of infection with either PAK or ΔFliC mutant. Results are representative of 3 independent experiments. *** *P*<0.001 when comparing with T0. ns  =  not significant.
(TIFF)

Figure S2 **Incubation of NCI-H292 cells with PAK and ΔFliC supernatant did not trigger a cytotoxic effect.** (A) Shows LDH levels (% of total LDH) in supernatants of NCI-H292 epithelial cells incubated with either PAK or ΔFliC supernatant. (B) Shows the levels of total proteins detected in adherent NCI-H292 epithelial cells, following stimulation with either PAK or ΔFliC supernatant. Results are representative of 3 independent experiments. * *P*<0.05 when comparing PAK to ΔFliC. ns  =  not significant.
(TIFF)

Figure S3 **Immuno-blotting analysis of IL-1β in NCI-H292 cells.** Cells were left unstimulated (NS) or stimulated with purified flagellin (Flag) at 1 µg/ml for 24 hours. Sixty µg of whole cell lysates were used for immunoblotting. Whole cell lysates from MH-S cells, macrophages cell line (Mac), stimulated with PAK at 1 MOI was used as a positive control (C). An anti-IL-1β polyclonal goat antibody from Santa Cruz was used at 1/200 overnight at 4°C. The second anti-goat polyclonal antibody was used at 1/20000 for one hour at room temperature. β-actine was used as a control for protein loading. The first anti-β-actine polyclonal antibody was purchased from Sigma and used at 1/20000 for one hour at room temperature. The anti-mouse second antibody was used at 1/20000 for one hour. Results are representative of 3 independent experiments. ns  =  not significant.
(TIFF)

## References

[1] Lau GW, Hassett DJ, Britigan BE (2005). Modulation of lung epithelial functions by Pseudomonas aeruginosa.. Trends Microbiol.

[2] Gomez MI, Prince A (2007). Opportunistic infections in lung disease: Pseudomonas infections in cystic fibrosis.. Curr Opin Pharmacol.

[3] Medzhitov R, Janeway CA (1997). Innate immunity: the virtues of a nonclonal system of recognition.. Cell.

[4] Mayer AK, Muehmer M, Mages J, Gueinzius K, Hess C (2007). Differential recognition of TLR-dependent microbial ligands in human bronchial epithelial cells.. J Immunol.

[5] Rivas-Santiago B, Juarez E (2007). [Toll-like receptor in lung response to pathogens].. Rev Invest Clin.

[6] Kipnis E, Sawa T, Wiener-Kronish J (2006). Targeting mechanisms of Pseudomonas aeruginosa pathogenesis.. Med Mal Infect.

[7] Ramphal R, Balloy V, Jyot J, Verma A, Si-Tahar M (2008). Control of Pseudomonas aeruginosa in the lung requires the recognition of either lipopolysaccharide or flagellin.. J Immunol.

[8] Hayashi F, Smith KD, Ozinsky A, Hawn TR, Yi EC (2001). The innate immune response to bacterial flagellin is mediated by Toll-like receptor 5.. Nature.

[9] Buchanan PJ, Ernst RK, Elborn JS, Schock B (2009). Role of CFTR, Pseudomonas aeruginosa and Toll-like receptors in cystic fibrosis lung inflammation.. Biochem Soc Trans.

[10] Geddes K, Magalhaes JG, Girardin SE (2009). Unleashing the therapeutic potential of NOD-like receptors.. Nat Rev Drug Discov.

[11] Wilmanski JM, Petnicki-Ocwieja T, Kobayashi KS (2008). NLR proteins: integral members of innate immunity and mediators of inflammatory diseases.. J Leukoc Biol.

[12] Vinzing M, Eitel J, Lippmann J, Hocke AC, Zahlten J (2008). NAIP and Ipaf control Legionella pneumophila replication in human cells.. J Immunol.

[13] Rose MC, Nickola TJ, Voynow JA (2001). Airway mucus obstruction: mucin glycoproteins, MUC gene regulation and goblet cell hyperplasia.. Am J Respir Cell Mol Biol.

[14] Basbaum C, Lemjabbar H, Longphre M, Li D, Gensch E (1999). Control of mucin transcription by diverse injury-induced signaling pathways.. Am J Respir Crit Care Med.

[15] Gendler SJ, Spicer AP (1995). Epithelial mucin genes.. Annu Rev Physiol.

[16] Rose MC, Voynow JA (2006). Respiratory tract mucin genes and mucin glycoproteins in health and disease.. Physiol Rev.

[17] Li JD (2003). Exploitation of host epithelial signaling networks by respiratory bacterial pathogens.. J Pharmacol Sci.

[18] Fahy JV, Dickey BF (2010). Airway mucus function and dysfunction.. N Engl J Med.

[19] Vestbo J, Lange P, Hansen EF (2002). [Chronic obstructive pulmonary disease. World COPD Day 2002].. Ugeskr Laeger.

[20] Ho SB, Niehans GA, Lyftogt C, Yan PS, Cherwitz DL (1993). Heterogeneity of mucin gene expression in normal and neoplastic tissues.. Cancer Res.

[21] Li JD, Dohrman AF, Gallup M, Miyata S, Gum JR (1997). Transcriptional activation of mucin by Pseudomonas aeruginosa lipopolysaccharide in the pathogenesis of cystic fibrosis lung disease.. Proc Natl Acad Sci U S A.

[22] Dohrman A, Miyata S, Gallup M, Li JD, Chapelin C (1998). Mucin gene (MUC 2 and MUC 5AC) upregulation by Gram-positive and Gram-negative bacteria.. Biochim Biophys Acta.

[23] Li JD, Feng W, Gallup M, Kim JH, Gum J (1998). Activation of NF-kappaB via a Src-dependent Ras-MAPK-pp90rsk pathway is required for Pseudomonas aeruginosa-induced mucin overproduction in epithelial cells.. Proc Natl Acad Sci U S A.

[24] Dif F, Wu YZ, Burgel PR, Ollero M, Leduc D (2010). Critical role of cytosolic phospholipase A2{alpha} in bronchial mucus hypersecretion in CFTR-deficient mice.. Eur Respir J.

[25] Balloy V, Verma A, Kuravi S, Si-Tahar M, Chignard M (2007). The role of flagellin versus motility in acute lung disease caused by Pseudomonas aeruginosa.. J Infect Dis.

[26] Song KS, Kim HJ, Kim K, Lee JG, Yoon JH (2009). Regulator of G-protein signaling 4 suppresses LPS-induced MUC5AC overproduction in the airway.. Am J Respir Cell Mol Biol.

[27] Sun Y, Karmakar M, Roy S, Ramadan RT, Williams SR (2010). TLR4 and TLR5 on corneal macrophages regulate Pseudomonas aeruginosa keratitis by signaling through MyD88-dependent and -independent pathways.. J Immunol.

[28] Molofsky AB, Byrne BG, Whitfield NN, Madigan CA, Fuse ET (2006). Cytosolic recognition of flagellin by mouse macrophages restricts Legionella pneumophila infection.. J Exp Med.

[29] Kofoed EM, Vance RE (2011). Innate immune recognition of bacterial ligands by NAIPs determines inflammasome specificity.. Nature.

[30] Zhao Y, Yang J, Shi J, Gong YN, Lu Q (2011). The NLRC4 inflammasome receptors for bacterial flagellin and type III secretion apparatus.. Nature.

[31] Raoust E, Balloy V, Garcia-Verdugo I, Touqui L, Ramphal R (2009). Pseudomonas aeruginosa LPS or flagellin are sufficient to activate TLR-dependent signaling in murine alveolar macrophages and airway epithelial cells.. PLoS One.

[32] Shanks KK, Guang W, Kim KC, Lillehoj EP (2010). Interleukin-8 production by human airway epithelial cells in response to Pseudomonas aeruginosa clinical isolates expressing type a or type b flagellins.. Clin Vaccine Immunol.

[33] Bautista MV, Chen Y, Ivanova VS, Rahimi MK, Watson AM (2009). IL-8 regulates mucin gene expression at the posttranscriptional level in lung epithelial cells.. J Immunol.

[34] Vargaftig BB, Singer M (2003). Leukotrienes, IL-13, and chemokines cooperate to induce BHR and mucus in allergic mouse lungs.. Am J Physiol Lung Cell Mol Physiol.

[35] Dasgupta N, Wolfgang MC, Goodman AL, Arora SK, Jyot J (2003). A four-tiered transcriptional regulatory circuit controls flagellar biogenesis in Pseudomonas aeruginosa.. Mol Microbiol.

[36] Verma A, Arora SK, Kuravi SK, Ramphal R (2005). Roles of specific amino acids in the N terminus of Pseudomonas aeruginosa flagellin and of flagellin glycosylation in the innate immune response.. Infect Immun.

[37] Garcia-Verdugo I, BenMohamed F, Tattermusch S, Leduc D, Charpigny J (2012). A role for 12R-lipooxygenase in MUC5AC expression by respiratory epithelial cells.. Eur Respir J. *In press*.

[38] Kim S, Lewis C, Nadel JA (2011). Epidermal growth factor receptor reactivation induced by E-prostanoid-3 receptor- and tumor necrosis factor-alpha-converting enzyme-dependent feedback exaggerates interleukin-8 production in airway cancer (NCI-H292) cells.. Exp Cell Res.

[39] Arora SK, Dasgupta N, Lory S, Ramphal R (2000). Identification of two distinct types of flagellar cap proteins, FliD, in Pseudomonas aeruginosa.. Infect Immun.

[40] Finzi L, Barbu V, Burgel PR, Mergey M, Kirkwood KS (2006). MUC5AC, a gel-forming mucin accumulating in gallstone disease, is overproduced via an epidermal growth factor receptor pathway in the human gallbladder.. Am J Pathol.

[41] Burgel PR, Lazarus SC, Tam DC, Ueki IF, Atabai K (2001). Human eosinophils induce mucin production in airway epithelial cells via epidermal growth factor receptor activation.. J Immunol.

