# Correction: A Crucial Role of Flagellin in the Induction of Airway Mucus Production by *Pseudomonas aeruginosa*

**DOI:** 10.1371/annotation/7718a413-90cf-4183-9eb0-3a1b7c5bcd83

**Published:** 2012-08-09

**Authors:** Fatima BenMohamed, Ignacio Garcia-Verdugo, Mathieu Medina, Viviane Balloy, Michel Chignard, Reuben Ramphal, Lhousseine Touqui

The first author's name was spelled incorrectly. The correct name is: Fatima BenMohamed. The correction citation is: BenMohamed F, Garcia-Verdugo I, Medina M, Balloy V, Chignard M, et al. (2012) A Crucial Role of Flagellin in the Induction of Airway Mucus Production by Pseudomonas aeruginosa. PLoS ONE 7(7): e39888. doi:10.1371/journal.pone.0039888 

