# Supplementary figures and images for: A Crucial Role of Flagellin in the Induction of Airway Mucus Production by Pseudomonas aeruginosa
Source: PLoS One. 2012 Jul 2;7(7):e39888. doi: 10.1371/journal.pone.0039888 (PMC3388098; doi:10.1371/journal.pone.0039888)

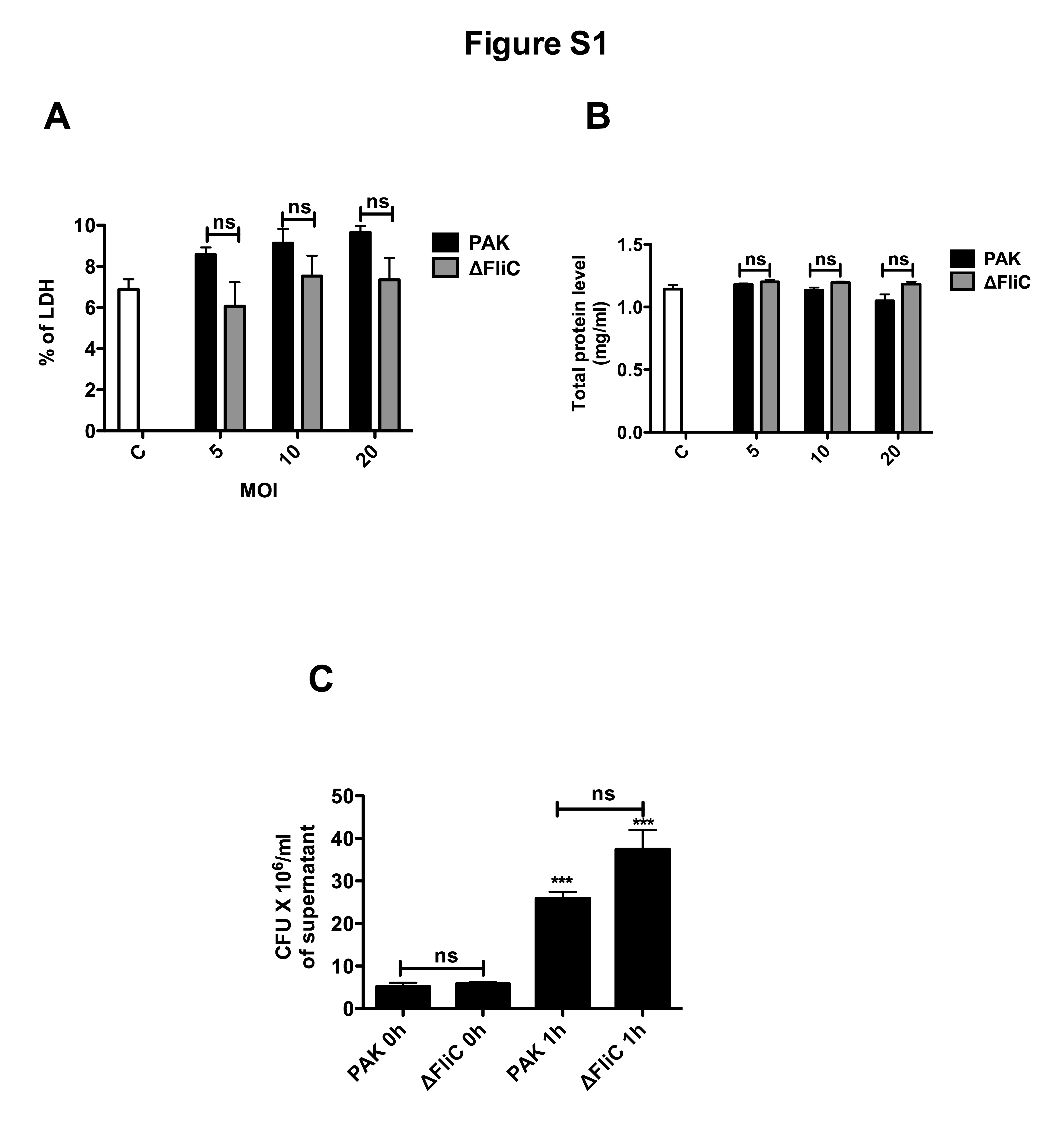

Supplement: Figure S1 — Infection of NCI-H292 cells with PAK and ΔFliC bacteria results in no cytotoxic effect. (A) Shows LDH level released in supernatants of NCI-H292 epithelial cells (determined as % of total cell LDH) following infection with either PAK or ΔFliC living bacteria. (B) Compares the level total proteins detected in adherent NCI-H292 epithelial cells following stimulation with either PAK or ΔFliC living bacteria. (C) Compares the amount of bacteria (CFU) detected in NCI-H292 cell supernatants after the first hour of infection with either PAK or ΔFliC mutant. Results are representative of 3 independent experiments. *** P<0.001 when comparing with T0. ns = not significant. (TIFF) [file pone.0039888.s001.tif]

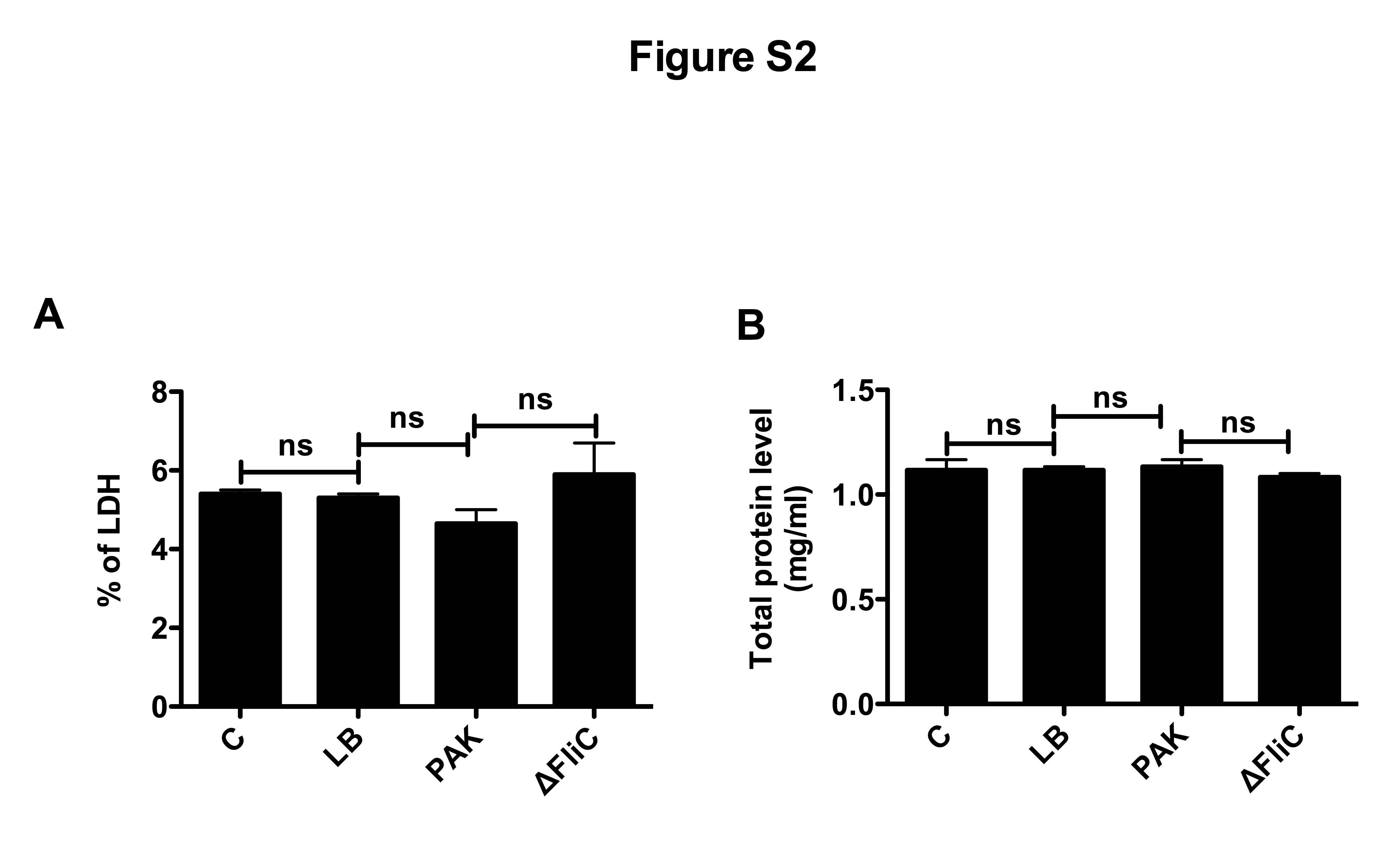

Supplement: Figure S2 — Incubation of NCI-H292 cells with PAK and ΔFliC supernatant did not trigger a cytotoxic effect. (A) Shows LDH levels (% of total LDH) in supernatants of NCI-H292 epithelial cells incubated with either PAK or ΔFliC supernatant. (B) Shows the levels of total proteins detected in adherent NCI-H292 epithelial cells, following stimulation with either PAK or ΔFliC supernatant. Results are representative of 3 independent experiments. * P<0.05 when comparing PAK to ΔFliC. ns = not significant. (TIFF) [file pone.0039888.s002.tif]

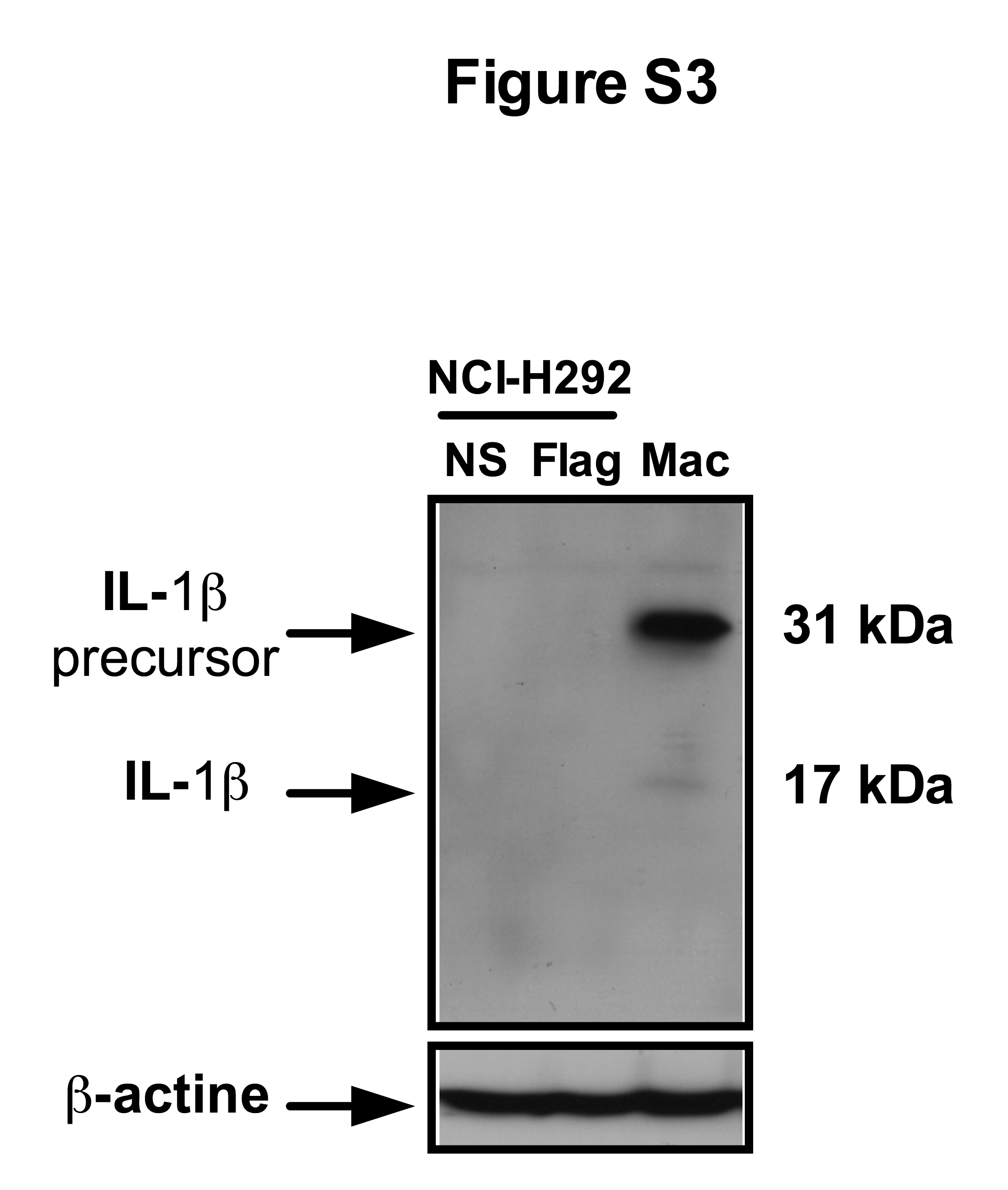

Supplement: Figure S3 — Immuno-blotting analysis of IL-1β in NCI-H292 cells. Cells were left unstimulated (NS) or stimulated with purified flagellin (Flag) at 1 µg/ml for 24 hours. Sixty µg of whole cell lysates were used for immunoblotting. Whole cell lysates from MH-S cells, macrophages cell line (Mac), stimulated with PAK at 1 MOI was used as a positive control (C). An anti-IL-1β polyclonal goat antibody from Santa Cruz was used at 1/200 overnight at 4°C. The second anti-goat polyclonal antibody was used at 1/20000 for one hour at room temperature. β-actine was used as a control for protein loading. The first anti-β-actine polyclonal antibody was purchased from Sigma and used at 1/20000 for one hour at room temperature. The anti-mouse second antibody was used at 1/20000 for one hour. Results are representative of 3 independent experiments. ns = not significant. (TIFF) [file pone.0039888.s003.tif]
